# A balanced measure shows superior performance of pseudobulk methods in single-cell RNA-sequencing analysis

**DOI:** 10.1038/s41467-022-35519-4

**Published:** 2022-12-22

**Authors:** Alan E. Murphy, Nathan G. Skene

**Affiliations:** 1 UK Dementia Research Institute at Imperial College London, London, W12 0BZ UK; 2 Department of Brain Sciences, Imperial College London, London, W12 0BZ UK

**Keywords:** Communication and replication, Statistical methods, Computational models, Bioinformatics, Computational science

**arising from** Zimmerman, K. D., Espeland, M. A. & Langefeld, C. D. *Nature Communications* 10.1038/s41467-021-21038-1 (2021)

Recently, Zimmerman et al.^1^, highlighted the importance of accounting for the dependence between cells from the same individual when conducting differential expression analysis on single-cell RNA-sequencing data. Their work proved the inadequacy of pseudoreplication approaches for such analysis—this was an important step forward that was conclusively proven by them. However, there appear to be limitations in both their benchmarking and simulation approaches. Here, we corrected these issues, reran the author’s analysis and found that pseudobulk methods outperformed mixed models. Based on these findings, we recommend the use of pseudobulk approaches for differential expression in single-cell RNA-sequencing analyses.

Zimmerman et al.^1^, performed a systematic analysis of differential expression methods’ type-1 error rates; the proportion of non-differentially expressed genes indicated as differentially expressed by a model. Their analysis was conducted on simulated, single-cell expression data across 20,000 iterations. The authors tested iterations of 5–40 individuals and 50–500 cells using an unadjusted *p*-value cut-off of 0.05 for significance. Plotting the results showed pseudobulk approaches had the lowest type-1 error at every iteration (Supplementary Fig. 1). However, evaluating such models on their type-1 or type-2 error rate in isolation is insufficient to determine their true performance. For example, a method with low type-1 error may have a high type-2 error rate. Therefore, we need to consider both type-1 and type-2 error rate to accurately benchmark the models. Moreover, because no seed was set for the pseudo-random number generator used in their hierarchical single-cell expression simulation approach (hierarchicell), the different methods evaluated by Zimmerman et al. were compared on different simulated datasets. Here, we modified Zimmerman et al.’s hierachicell approach to simulate both differentially expressed and non-differentially expressed genes. The differentially expressed genes were randomly simulated with a fold change between 1.1 and 10. We further modified hierachicell to correct the seeding of the pseudo-random number generator to enable fair comparisons across models.

We tested the models using the Matthews Correlation Coefficient (MCC) giving a balanced measure of performance. MCC is a well-known and frequently adopted metric in the machine learning field, which offers a more informative and reliable score on binary classification problems^2^. MCC produces scores in [−1,1] and will only assign a high score if a model performs well on both non-differentially and differentially expressed genes. Moreover, MCC scores are proportional to both the size of the differentially and non-differentially expressed genes, so it is robust to imbalanced datasets. We also benchmarked the models using receiver operating characteristics (ROC) curves for different proportions of differentially expressed genes.

Our MCC analysis demonstrates that pseudobulk approaches achieve highest performance across individuals and cells variations (Fig. 1). There is one exception for sum pseudobulk, which performs worse than Tobit at 5 individuals and 10 cells. Figure 1 also highlights a trend whereby pseudoreplication models; ‘Modified t’, ‘Tobit’, ‘Two-part hurdle: Default’ and ‘Two-part hurdle: Corrected’ (which take cells as independent replicates) showed increasingly poor performance as the number of cells increases. This trend is likely due to the overestimation of power driven by the dependence between cells from the same individual^3^ and agrees with Zimmerman et al.’s findings^1^. On the other hand, both pseudobulk approaches; ‘Pseudobulk: Mean’ and ‘Pseudobulk: Sum’, showed improved performance as the number of cells increases. This trend was also noted in two of the other models; ‘GEE1’ and ‘Tweedie: GLMM’.

**Fig. 1:**
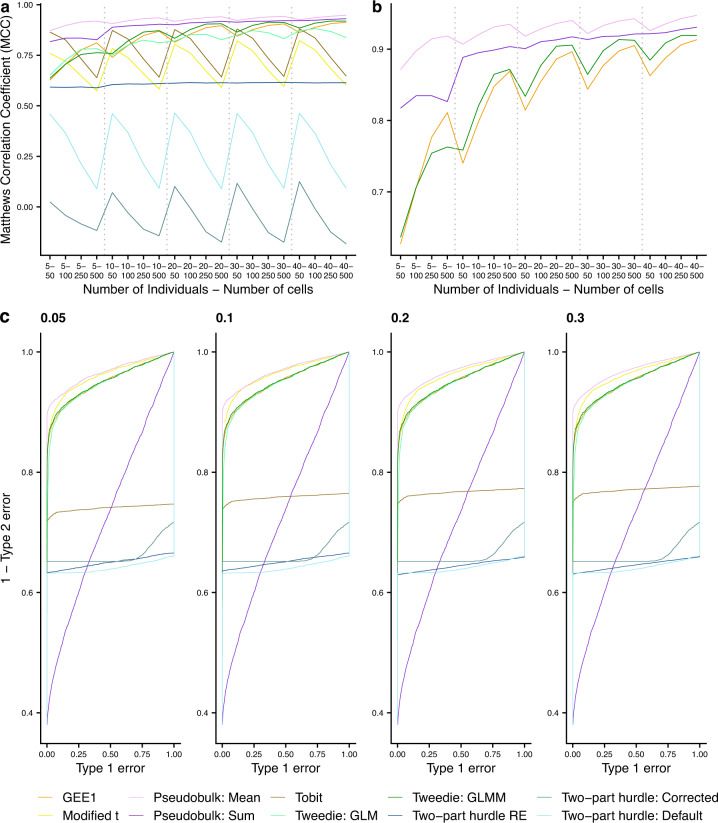
Performance of the analysed models. **a**, **b** give the average Matthews correlation coefficient from the 20,000 iterations; 50 runs for each of the 5–40 individuals and 50–500 cells at a *p*-value cut-off of 0.05 on 10,000 genes. **a** shows all benchmarked models whereas **b** focuses on the top four approaches. **c** gives the receiver operating characteristics (ROC) curve across 50 runs each for different proportions of simulated differentially expressed genes (DEGs)—0.05, 0.1, 0.2, 0.3. Twenty individuals were simulated for case and controls, each with 100 cells. The performance split by each iteration is given in Supplementary Table 2. The different models are pseudoreplication approaches; ‘Modified t’, ‘Tobit’, ‘Two-part hurdle: Default’, ‘Two-part hurdle: Corrected’, ‘GEE1’, ‘Tweedie: GLM’, pseudobulk approaches; ‘Pseudobulk: Mean’, ‘Pseudobulk: Sum’ and mixed model approaches; ‘Tweedie: GLMM’ and ‘Two-part hurdle: RE’. More detail on these models is given in Supplementary Table 1. Source data are provided as a Source Data file.

Moreover, for statistical test comparisons, another approach is to compare the power of tests at the same test size. That is, to compare the models’ sensitivity (1–type-2 error) at a consistent type-1 error rate. Therefore, we generated ROC curves for the different approaches, enabling such comparisons. For example, in Supplementary Fig. 2, we highlight the different sensitivity scores (1–type-2 error) of the models obtained at a consistent type-1 error rate of 0.05. We find that pseudobulk mean performs best at this type-1 error rate (with a sensitivity >0.9, whereas all other methods had <0.9) and at all other type-1 error rates (Fig. 1). Interestingly, we show that the two mixed model approaches (‘Two-part hurdle: RE’ and ‘GLMM Tweedie’) perform relatively poorly even compared to some pseudoreplication approaches. This analysis demonstrates how pseudobulk mean obtains low type-2 error rates, even at the lowest type-1 error rates of the methods benchmarked, supporting our MCC results.

Zimmerman et al. argued that pseudobulk methods are “overly conservative” relative to mixed models in their work. Specifically, they refer to pseudobulk approaches’ lower than nominal levels of type-1 error rates, demonstrated in their results where, based on a consistent *p*-value cut-off of 0.05, they benchmark the performance of different methods at identifying non-differentially expressed genes (Supplementary Fig. 1). Their analysis showed pseudobulk approaches’ type-1 error rates were below the expected 0.05 of false positives at each number of individuals and number of cells combination. In this analysis, it is true that pseudobulk approaches have mis-calibrated confidence intervals, obtaining fewer false positives than expected at a 0.05 *p*-value cut-off. Given this conservative 95% confidence intervals of pseudobulk methods, they could, as a result, have a higher type-2 error than other methods. However, our ROC analysis disproves this. It shows how, at equal type-1 error rates, pseudobulk mean has the lowest type-2 error rate of all tested methods (Fig. 1, Supplementary Fig. 2).

All analysis to this point have been on simulations with an equal number of cells in each sample. However, in real datasets this would never be the case^4^. To mirror this, we simulated data with an imbalanced number of cells between case and controls. We find that pseudobulk mean outperforms all other approaches on this analysis (Supplementary Fig. 3). The pseudobulk approach which aggregated by averaging rather than taking the sum appears to be the top performing overall. However, it is worth noting that hierarchicell does not normalise the simulated datasets before passing to the pseudobulk approaches. This is a standard step in such analysis to account for differences in sequencing depth and library sizes^5^. This approach was taken by Zimmerman et al. as their data are simulated one independent gene at a time without considering differences in library size. The effect of this step is more apparent on the imbalanced number of cells where pseudobulk sum’s performance degrades dramatically. Pseudobulk mean appears invariant to this missing normalisation step because of the averaging’s own normalisation effect. Importantly, this was a flaw in the simulation software strategy and does not show an improved performance of pseudobulk mean over sum. We believe this approach also affected the performance of pseudobulk sum on the different proportions of differentially expressed genes (Fig. 1).

Pseudobulk approaches were also found to be top performing in a recent review by Squair et al.,^6^. Notably, the pseudobulk method used here, DESeq2^5^, performed worse than other pseudobulk models in Squair et al.,’s analysis and so their adoption may further increase the performance of pseudobulk approaches on our dataset. Conversely, Squair et al., did not consider all models included in our analysis or the different forms of pseudobulk aggregation. Therefore, our results on sum and mean pseudobulk extend their findings and indicate that mean aggregation may be the best performing. However, the reader should be cognisant that the lack of a normalisation step based on the flaw in the simulation software strategy likely causes the increased performance of mean over sum aggregation. Further, the use of simulated datasets in our analysis may not accurately reflect the differences between individuals that are present in biological datasets. Thus, despite both our results and those reported by Squair et al., there is still room for further analysis, benchmarking more models, including different combinations of pseudobulk aggregation methods and models, on more representative simulated datasets and biological datasets to identify the optimal approach. Specifically, we would expect pseudobulk sum with a normalisation step to outperform pseudobulk mean since it can account for the intra-individual variance which is otherwise lost with pseudobulk mean, but this should be tested, including on imbalanced datasets and at consistent type-1 error rates.

In conclusion, our results demonstrate that pseudobulk approaches lead to the best performance for the analysis of single-cell expression data based on power at equivalent type-1 error rates and on MCC for both balanced and imbalanced number of cells, from this simulated dataset.

## Reporting summary

Further information on research design is available in the Nature Portfolio Reporting Summary linked to this article.

## Supplementary information

Supplementary Information

Reporting Summary

## Data Availability

The data underlying Fig. 1 (Bottom) and Supplementary Fig. 2 are available at https://github.com/Al-Murphy/reanalysis_scRNA_seq_benchmark (DOI^7^). All other relevant data supporting the key findings of this study are available within the article and its Supplementary Information files or from the corresponding author upon reasonable request. Source data are provided with this paper.

## Source data

Source Data

## Code availability

The modified version of hierarchicell which returns the Matthews correlation coefficient performance metric as well as the type-1 error rates, uses the same simulated data across approaches and has checkpointing capabilities (so runs can continue from where they left off if aborted or crashed) is available at: https://github.com/neurogenomics/hierarchicell (DOI^8^). The benchmarking script along with the results is available at: https://github.com/Al-Murphy/reanalysis_scRNA_seq_benchmark (DOI^7^).
