# Supplementary material for: A balanced measure shows superior performance of pseudobulk methods in single-cell RNA-sequencing analysis
Source: Nat Commun. 2022 Dec 22;13:7851. doi: 10.1038/s41467-022-35519-4 (PMC9780232; doi:10.1038/s41467-022-35519-4)
Supplement: Supplementary file 1 — Supplementary Information [file 41467_2022_35519_MOESM1_ESM.pdf]

A balanced measure shows superior performance of pseudobulk methods in single-cell RNA-sequencing analysis

Murphy *et al.*

Supplementary Figures and Tables

Supplementary Figures

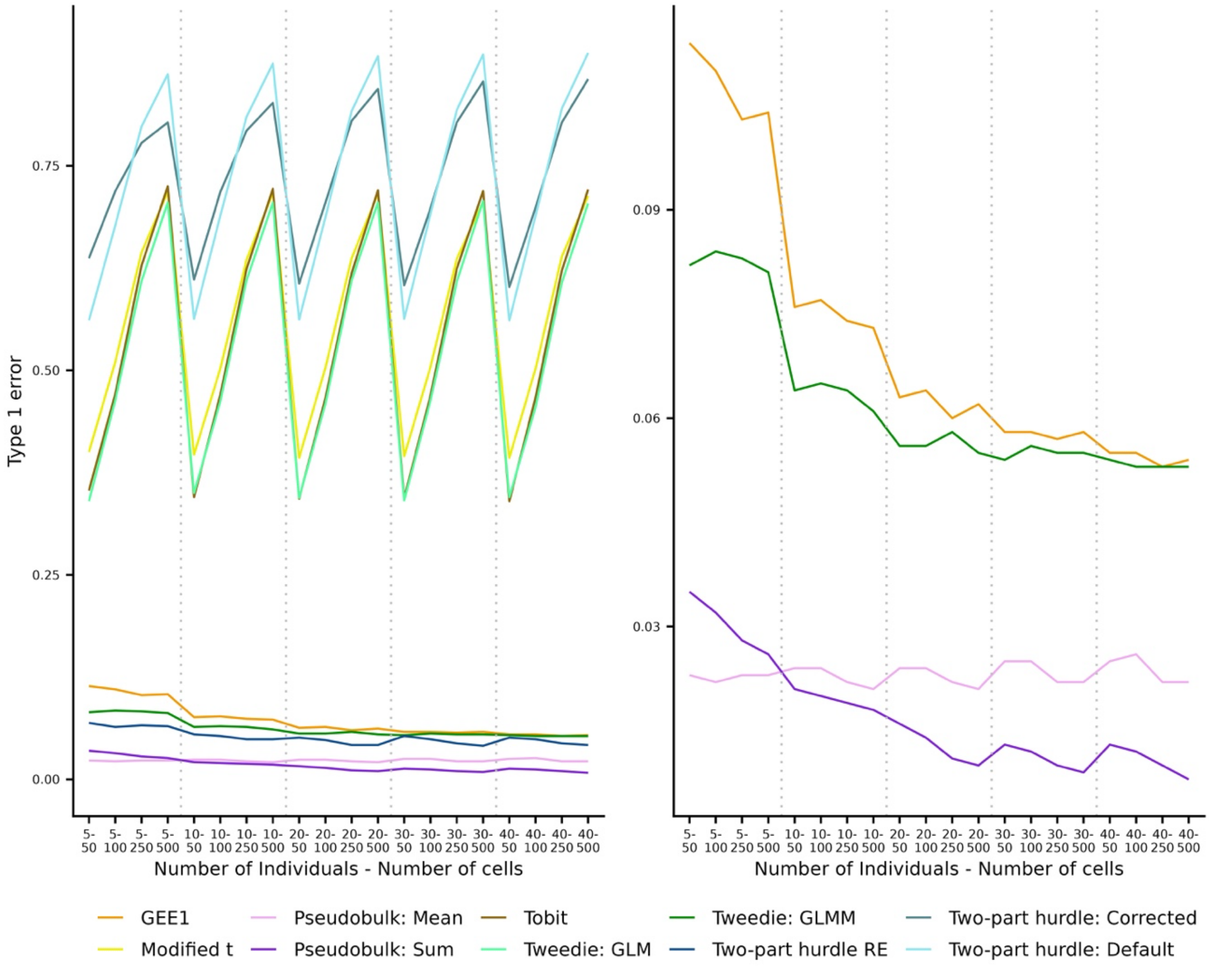

***Supplementary Figure 1**: The average type 1 error from the 20,000 iterations; 50 runs for each of the 5 to 40 individuals and 50 to 500 cells at a p-value cut-off of 0.05 on 5,000 genes reported by Zimmerman et al.[1]. Left shows all benchmarked models whereas right focuses on the top four approaches. The different models are pseudoreplication approaches; 'Modified t', 'Tobit', 'Two-part hurdle: Default', 'Two-part hurdle: Corrected', 'GEE1', 'Tweedie: GLM', pseudobulk approaches; 'Pseudobulk: Mean', 'Pseudobulk: Sum' and mixed model approaches; 'Tweedie: GLMM' and 'Two-part hurdle: RE'. Source data are provided in the as a source data file and from the original publication[1].*

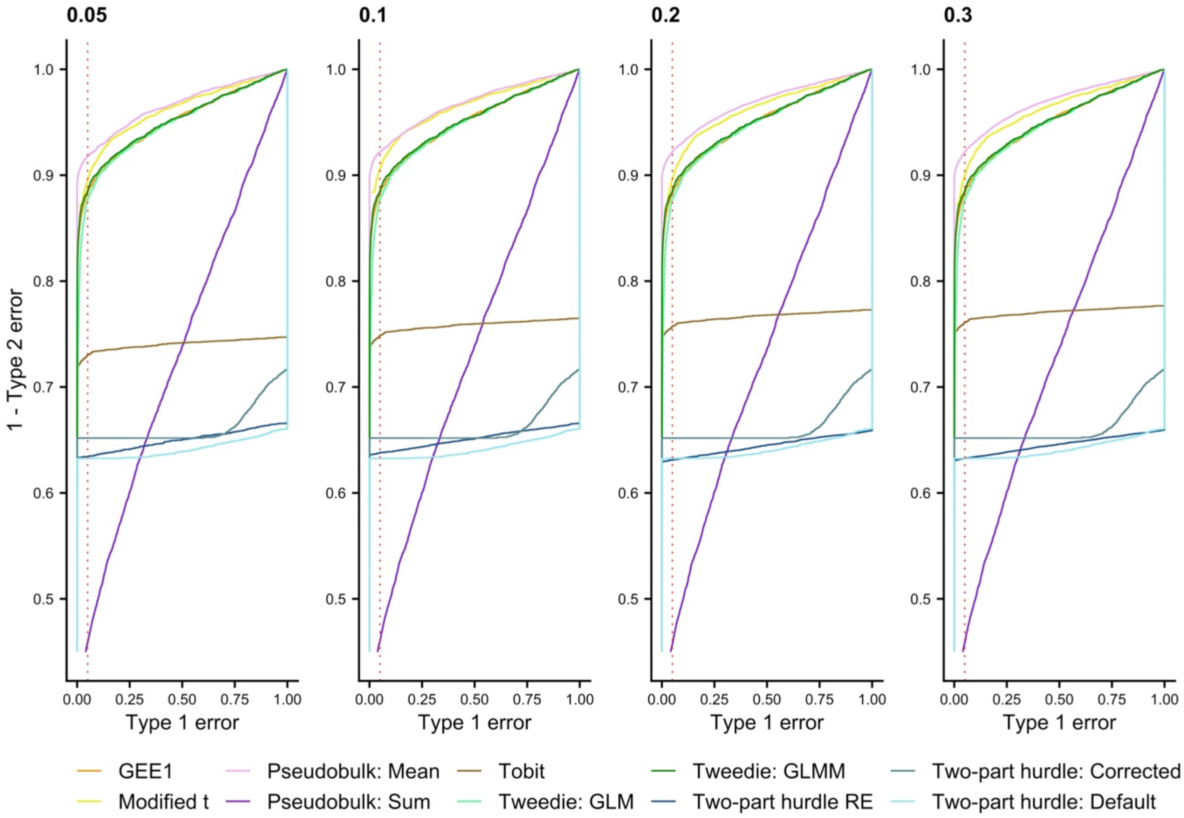

Supplementary Figure 2*: Pseudobulk mean is best performing at a constant type 1 error rate. The four images give the receiver operating chracteristics (ROC) curve across 50 runs each for different proportions of simulated differentially expressed genes (DEGs) - 0.05, 0.1,0.2,0.3. 20 individuals were simulated for case and controls, each with 100 cells. The proportion of DEGs are of the 5,000 non-DEGs simulated. The sensitivity (1 – type 2 error) of the different approaches at a 0.05 type 1 error value are highlighted by the red dashed line. The different models are pseudoreplication approaches; 'Modified t', 'Tobit', 'Two-part hurdle: Default', 'Two-part hurdle: Corrected', 'GEE1', 'Tweedie: GLM', pseudobulk approaches; 'Pseudobulk: Mean', 'Pseudobulk: Sum' and mixed model approaches; 'Tweedie: GLMM' and 'Two-part hurdle: RE'. Source data are provided[2].*

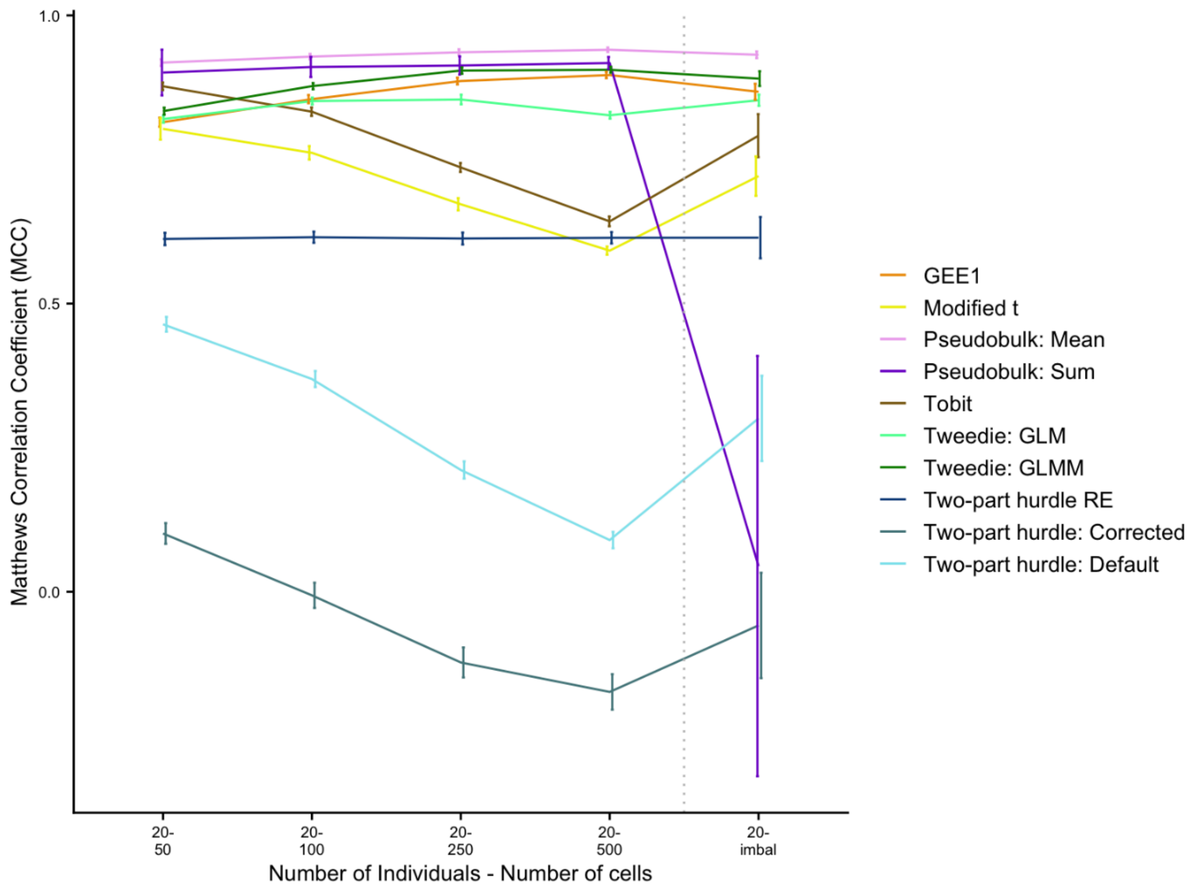

***Supplementary Figure 3**: The average Matthews correlation coefficient of all benchmarked models across all balanced number of cells and the imbalanced number of cells for 20 individuals; 50 runs for each at a p-value cut-off of 0.05 on 5,000 genes. The number of cells were randomly chosen using a gamma distribution with shape 4 and scale 45 separately for cases and controls to produce the imbalanced dataset (giving a mean 150-200 cells). The error bars give 1 standard deviation around the mean. The different models are pseudoreplication approaches; 'Modified t', 'Tobit', 'Two-part hurdle: Default', 'Two-part hurdle: Corrected', 'GEE1', 'Tweedie: GLM', pseudobulk approaches; 'Pseudobulk: Mean', 'Pseudobulk: Sum' and mixed model approaches; 'Tweedie: GLMM' and 'Two-part hurdle: RE'. Source data are provided as a Source Data file.*

Supplementary Tables

Method	Description	Method Type	Implementation
GEE1	Generalised linear models - generalized estimating equation	Pseudoreplication	geepack
Modified t	Reproducibility-Optimized Statistical Testings (ROTS)	Pseudoreplication	ROTS
Pseudobulk: Mean	Pseudobulk with mean aggregation	Pseudobulk	DESeq2
Pseudobulk: Sum	Pseudobulk with sum aggregation	Pseudobulk	DESeq2
Tobit	Monocole	Pseudoreplication	Monocole
Tweedie: GLM	Generalised linear model with Tweedie distribution.	Pseudoreplication	glmmTMB
Tweedie: GLMM	Generalised linear mixed-effects model with Tweedie distribution.	Mixed Model	glmmTMB
Two-part hurdle RE	Model-based analysis of single-cell transcriptomics (MAST) with a random effect for individuals.	Mixed Model	MAST
Two-part hurdle: Corrected	MAST batch-corrected for individuals.	Pseudoreplication	MAST & ComBat
Two-part hurdle: Default	MAST without random effects.	Pseudoreplication	MAST

Supplementary Table 1: *The different methods benchmarked in the analysis with their implementation approach and methodological types.*

**Method**	**0.05**	**0.1**	**0.2**	**0.3**
GEE1	0.950138	0.950138	0.950138	0.950138
Modified t	0.956821	0.960299	0.957801	0.957978
Pseudobulk: Mean	0.965886	0.967704	0.968249	0.968664
Pseudobulk: Sum	0.731316	0.731252	0.730117	0.729725
Tobit	0.740201	0.758228	0.76654	0.770334
Tweedie: GLM	0.947437	0.947437	0.947437	0.947437
Tweedie: GLMM	0.951053	0.951053	0.951053	0.951053
Two-part hurdle RE	0.649905	0.651323	0.644665	0.645651
Two-part hurdle: Corrected	0.662429	0.662429	0.662429	0.662429
Two-part hurdle: Default	0.641707	0.641707	0.641707	0.641707

Supplementary Table 2*: The area under the receiver operating characteristics curve (AUC) of all benchmarked models across different proportions of simulated differentially expressed genes (DEGs) - 0.05, 0.1,0.2,0.3. 20 individuals were simulated for case and controls, each with 100 cells. The proportion of DEGs are of the 5,000 non-DEGs simulated. The values are rounded to six decimal places. The different models are pseudoreplication approaches; 'Modified t', 'Tobit', 'Two-part hurdle: Default', 'Two-part hurdle: Corrected', 'GEE1', 'Tweedie: GLM', pseudobulk approaches; 'Pseudobulk: Mean', 'Pseudobulk: Sum' and mixed model approaches; 'Tweedie: GLMM' and 'Two-part hurdle: RE'.*

References

1. Zimmerman, K. D., Espeland, M. A. & Langefeld, C. D. A practical solution to pseudoreplication bias in single-cell studies. *Nat. Commun.* **12**, 738 (2021).
2. Murphy, A. *Al-Murphy/reanalysis_scRNA_seq_benchmark: Reanalysis scRNA-seq benchmark*. (Zenodo, 2022). doi:10.5281/zenodo.7356243.
